# Lemierre's Syndrome Under the Disguise of COVID-19 Pneumonia: A Case Report and Systematic Review

**DOI:** 10.7759/cureus.45827

**Published:** 2023-09-23

**Authors:** Yani Zhang, Yuhao Zeng

**Affiliations:** 1 Internal Medicine, MedStar Union Memorial Hospital, Baltimore, USA; 2 Internal Medicine, Cleveland Clinic Akron General, Akron, USA

**Keywords:** lemierre's and lemierre's-like syndrome, septic emboli, case report, fusobacterium thrombophlebitis, covid-19 pneumonia

## Abstract

Lemierre's syndrome refers to septic thrombophlebitis caused by *Fusobacterium necrophorum *bacteremia. The incidence of Lemierre's syndrome has risen over the past two decades. This report describes a case of a 31-year-old woman presenting with multifocal pneumonia and uncomplicated parapneumonic effusion, considered as COVID-19 pneumonia initially, then found to have Lemierre's syndrome with *Fusobacterium necrophorum* bacteremia and right internal jugular vein thrombophlebitis. Her condition improved after four weeks of antibiotics without anticoagulation. The article summarized the history, epidemiology, clinical presentation, differential diagnosis, and treatment of Lemierre's syndrome, the rare but severe complication of bacterial infection. The article also summarized six reported Lemierre's syndrome cases during the COVID-19 pandemic to emphasize the significance of having a broad differential diagnosis for respiratory symptoms, especially in the COVID-19 era.

## Introduction

Lemierre's syndrome refers to septic thrombophlebitis of the internal jugular vein caused by systemic bacteremia, most commonly *Fusobacterium necrophorum* bacteremia. The incidence of Lemierre's syndrome has risen over the past two decades. This report describes a case of a 31-year-old woman presenting with multifocal pneumonia and uncomplicated parapneumonic effusion, considered as COVID-19 pneumonia initially, then found to have Lemierre's syndrome with *Fusobacterium necrophorum* bacteremia and right internal jugular vein thrombophlebitis. Her condition improved after four weeks of antibiotics without anticoagulation. This article summarized the history, epidemiology, clinical presentation, differential diagnosis, and treatment of Lemierre's syndrome, the rare but severe complication of bacterial infection. The article also summarized six reported Lemierre's syndrome cases during the COVID-19 pandemic to emphasize the significance of having a broad differential diagnosis for respiratory symptoms, especially in the COVID-19 era.

## Case presentation

A 31-year-old female presented to the emergency department with 48 hours of sore throat and fever (38.2 C). Past medical history was notable for prior COVID-19 infection (two months ago), but nasal swab SARS-CoV-2 was not detected by polymerase chain reaction (PCR) for the current presentation. Enlarged tonsils with exudate and anterior cervical lymphadenopathy were noted on the exam. Rapid antigen detection test for *Streptococcus pyogenes (S. pyogenes)* was negative. She was discharged home with a short course of prednisone for a presumptive diagnosis of infectious mononucleosis and viscous lidocaine for symptom management. 

The patient's symptoms initially improved and then worsened, prompting a return to the ED via emergency medical services (EMS) seven days later. Upon arrival, she was tachycardic, and tachypneic, and required a 2L nasal cannula to maintain 90% oxygenation saturation. In addition to the sore throat, the patient had additional complaints of severe substernal chest pain, cough, intractable vomiting, and diarrhea. Chest pain was described as substernal, worsened with touch and deep inspiration, relieved by leaning forward. Initial labs (Table 1) were noted for thrombocytopenia, elevated D-dimer, and elevated N-terminal pro b-type natriuretic peptide (NT pro-BNP). Chest CT ruled out pulmonary embolism but showed multifocal pneumonia with small bilateral pleural effusions. Extensive viral workups (Table 2), including Epstein-Barr virus, cytomegalovirus, and repeated SARS-CoV-2, were all negative. While at ED, she was empirically treated for severe community-acquired pneumonia with doxycycline and ceftriaxone, then switched to azithromycin and ceftriaxone when admitted to the medical floor. However, in the afternoon, her pneumonia was worsening; therefore, antibiotics were broadened to vancomycin, piperacillin-tazobactam, and levofloxacin (atypical pneumonia coverage). On the same day evening, her blood cultures preliminarily resulted back as gram-negative bacilli; then, we tailored the regimen to piperacillin-tazobactam.

**Table 1:** Laboratory results on admission *Pathologist interpretation: WBC differential indicative of thrombocytopenia; few hypersegmented neutrophils and neutrophils with Dohle bodies present; mild lymphopenia BUN - blood urea nirtogen, AST - aspartate aminotransferase, ALT - alanine transaminase, NT pro-BNP - N-terminal pro b-type natriuretic peptide

Parameter	Value	Reference range
BUN	18 mg/dL	7-21
Creatinine	0.63 mg/dL	0.58-0.96
Bilirubin, total	2.2 mg/dL	0.2-1.3
Bilirubin, conjug	1.8 mg/dL	<0.2
AST	19 U/L	13-35
ALT	24 U/L	7-38
Alkaline Phosphatase	225 U/L	34-123
NT pro-BNP	624 pg/mL	<125
WBC*	9.91 k/uL	3.70-11.00
Hemoglobin	12.0 g/dl	11.5-15.5
Platelet	39 k/uL	150-400
D-Dimer	2800 ng/mL	<500
Fibrinogen	448 mg/dL	200-400
Procalcitonin	9.58 ng/dL	<0.09

**Table 2:** Microbiology result *Interpretation: no acute infection PCR - polymerase chain reaction, CMV - cytomegalovirus, AB - antibody, EVB - Epstein-Barr virus, AFB - acid-fast bacteria

Parameter	Specimen	Value
SARS-CoV-2	Upper respiratory tract swab	Not detected by PCR
Legionella AG urine	Urine	Negative
Hepatitis C AB IA	Blood	Negative
HIV 1/2 combo (AG/AB)	Blood	Negative
*Mycoplasma pneumoniae* IgM	Blood	Negative
CMV IgM AB	Blood	<0.8 (Negative)
Epstein-Barr panel*	Blood	EVB Vca IgG: 6.9 (Positive)
		EVB Vca IgM: 0.4 (Negative)
		EVB Ea antibody: 0.3 (Negative)
		EVB Na antibody: >0.8 (Positive)
*Staphylococcus aureus* PCR	Nasal	Negative
Fungal culture	Pleural fluid	Negative
AFB culture + stain	Pleural fluid	Negative

**Figure 1:**
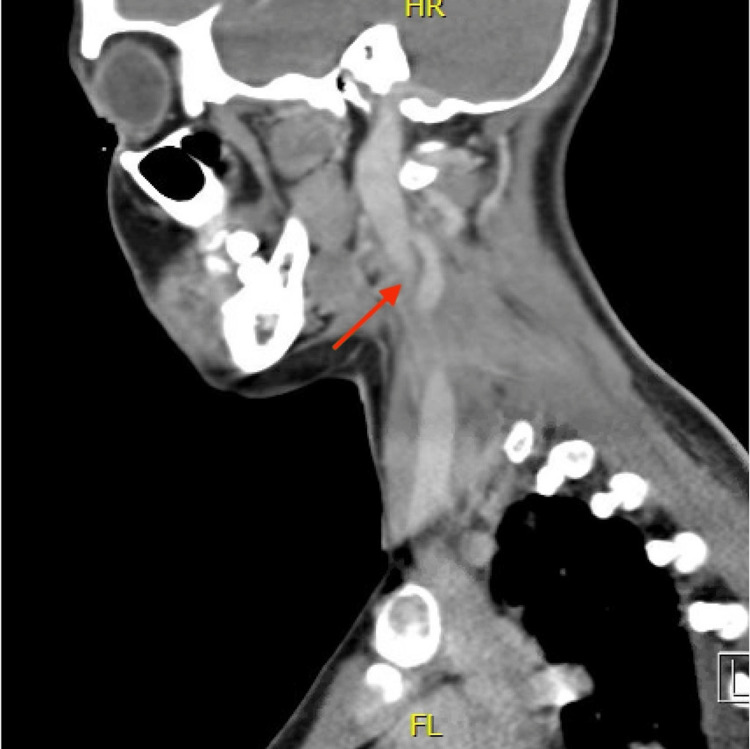
CT head (sagittal) Filling defect consistent with acute thrombophlebitis of the right internal jugular vein extending from C5-6 level to C3 level (red arrow)

**Figure 2:**
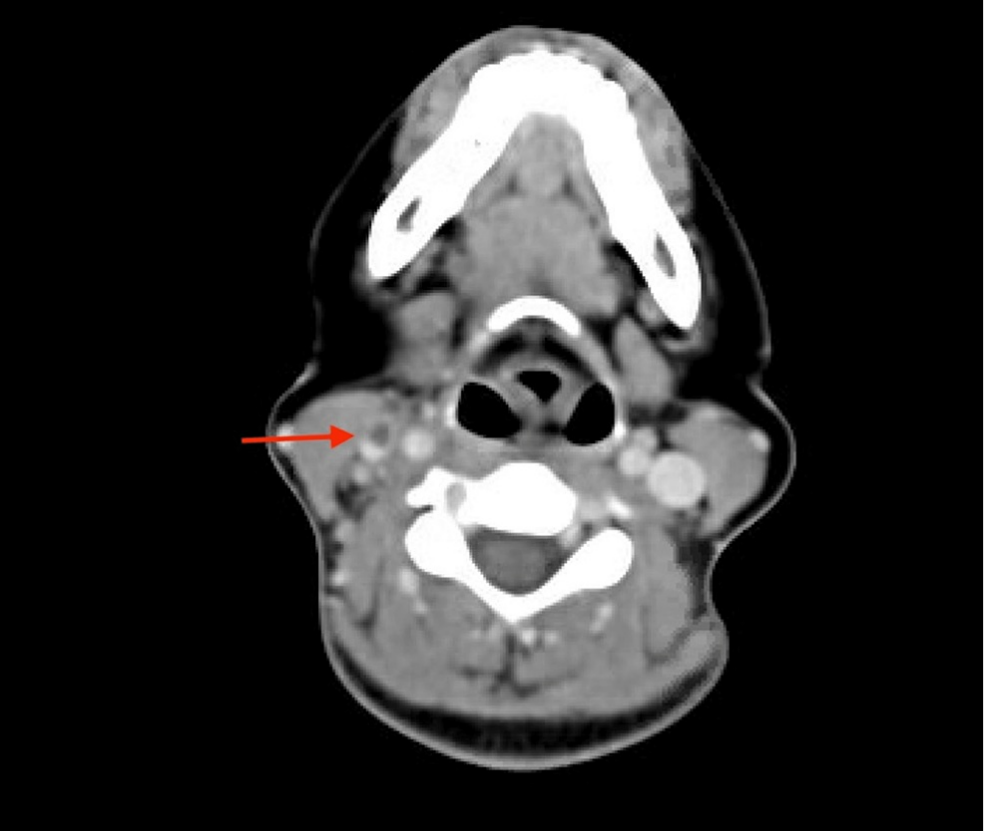
CT head (axial) Filling defect consistent with acute thrombophlebitis of the right internal jugular vein (red arrow). There is an asymmetric appearance of the right palatine tonsil pillar without evidence of a well-defined tonsillar abscess. Retropharyngeal edematous changes at the level of the oropharynx, and hypopharynx extending caudally to the supraglottic level

**Figure 3:**
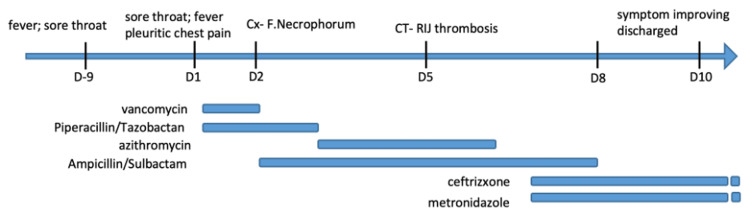
Hospitalization course and antibiotics regimen F. necrophorum - Fusobacterium necrophorum, RIJ - right internal jugula

On hospital day four, blood cultures drawn on presentation returned as *Fusobacterium necrophorum*. A neck ultrasound was performed to assess for suspected Lemierre's syndrome but showed normal jugular venous compressibility and venous waveform. At this time, the patient still had a persistent sore throat, no lateral neck pain, and no posterior pain, which yielded low suspicion of discitis or osteomyelitis. Since Lemirerre's syndrome is high on the differential diagnosis list at this moment, a CT neck was ordered. The next day, CT neck with contrast confirmed a thrombus in the right internal jugular vein from C3 to C6, with no evidence of a well-defined tonsillar abscess (Figure 1). Retropharyngeal edematous changes in the region adjacent to the thrombus are suggestive of original tonsillitis having progressed to retropharyngeal space infection (Figure 2). A thoracentesis revealed 450ml of straw-yellow fluid and confirmed uncomplicated parapneumonic exudative pleural effusion (Table 3), considered generated from pulmonary septic embolization. Her hospital course and treatment are summarized below (Figure 3).

**Table 3:** Pleural fluid analysis RBC - red blood cells, BF - body fluid, LDH - lactate dehydrogenase *BF pathologist comments: the specimen consists of neutrophils, macrophages/monocytes, lymphocytes, and occasional lymphoid cells with moderately abundant cytoplasm, consistent with reactive lymphocytes and rare plasma cells †Positive for exudative effusion based on Light's criteria; BF/serum protein ratio = 3.06 > 0.6; BF/serum LDH ratio = 0.53 > 0.5

Parameter	Value	Reference range
Color	Yellow	N/A
Clarity	Clear	N/A
RBC, BF	<2000 /ul	<2000
Total nucleated cells, BF	600 /ul	<1000
Neut%, BF	71%	0-1%
Lymph%, BF	9%	18-36%
Mono%, BF	2%	N/A
Macro%, BF	13%	64-80%
pH, BF	7.41	N/A
LDH, BF^†^	545 U/L	N/A
Protein, BF^†^	3.5 g/dL	N/A
Glucose, BF	70 mg/dL	N/A
Cholesterol, BF	113 mg/dL	N/A

Her fever resolved since hospital day five and the rest of her symptoms slowly improved. The patient was discharged on hospital day 10 with a plan of a four-week course of ceftriaxone and metronidazole. With continued outpatient follow-up, she became symptom-free, returned to daily activity, and her C-reactive protein (CRP; 0.6 mg/dL from peak 12.0 mg/dL) and erythrocyte sedimentation rate (ESR; 12 mm/hr from peak 114 mm/hr)were back to normal in nearly one month.

## Discussion

Lemierre's syndrome was first reported by Lemierre during a time when its characteristic symptoms were more commonly observed due to the lack of antibiotics. In the modern era, it can be hard to recognize this relatively rare syndrome because of the widespread use of antibiotics and the lack of awareness among many clinicians of this potentially life-threatening condition. 

Anaerobic organisms, most commonly *Fusobacterium necrophorum (F. necrophorum)*, are responsible for the majority of bacteremia in Lemierre's syndrome. *F. necrophorum* is a gram-negative anaerobic rod as part of the normal oral flora. Typically, it does not invade mucosal membranes and cause bacteremia; however, the risk of invasive infection will increase if there is mucosal inflammation or infection caused by a virus or bacteria, as illustrated in sporadic cases of Lemierre's syndrome following infectious mononucleosis.

The most common presenting symptom of Lemierre's syndrome is sore throat accompanied by other symptoms, including neck pain, neck mass, pleuritic chest pain, dyspnea, and cough. Clinical manifestations varied depending on the extent of metastatic disease. In this case, the patient initially presented with a sore throat, which initially improved with the treatment of infectious mononucleosis. Then, later, she developed worsening tachypnea, dyspnea, desaturation, and pleuritic chest pain, indicating pulmonary metastatic infection. As pulmonary symptoms and a recent history of pharyngitis are common in patients with Lemierre's syndrome, clinicians should consider this uncommon but possible differential diagnosis.

There are two solid pieces of evidence for the proper diagnosis: identification of internal jugular vein (IJV) thrombophlebitis and blood culture positivity. When patients exhibit symptoms of pharyngitis and fever, cervical ultrasound or contrast-enhanced computed tomography (CT) of the neck/chest is typically ordered to rule out deep cervical tissue infection. A systematic review suggested CT was the most requested scan in 55% of cases of Lemierre's syndrome, followed by ultrasound in 26% of cases, and magnetic resonance imaging in 6%, either alone or in combination [1]. Ultrasonography is less sensitive than CT and less useful for the evaluation of deep regions below the clavicle or mandible. Therefore, many physicians recommended contrast-enhanced CT as the preferred imaging study [2]. In the majority of reported cases, blood culture grew anaerobic bacteria, especially *F. necrophorum*, with an average blood culture time-to-positivity of 15 hours [3]. Europe Lemierre Study Group suggested maintaining a high level of suspicion for this rare illness when patients presented with fever, sore throat, and unilateral neck tenderness, especially when rapid streptococcal antigen is negative and multifocal pneumonia is observed on a chest X-ray [4].

Other mimics include infectious mononucleosis, community-acquired pneumonia, and pulmonary embolism. Nowadays, COVID-19 has also become one of the significant differential diagnoses, and any patient presenting with fever and sore throat symptoms was presumed in COVID-19 infection until proven negative, which would require intensive SARS-CoV-2 tests and prolonged isolation, further delaying diagnosis. A literature review was conducted on published case reports and series related to Lemierre's syndrome using the keywords "Lemierre" or "Lemierre syndrome" in the PubMed database. In the 18 months following the declaration of the pandemic by the World Health Organization (WHO) on March 11, 2021, six cases were reported. Based on these case reports, the initial diagnosis, confirmatory tests, and duration since admission were compiled (Table 4). All six cases [5-10] were initially considered as COVID-19 pneumonia based on presenting symptoms, followed by multiple negative COVID-19 tests. All cases were diagnosed between four and seven days after hospitalization. Four out of six cases received anticoagulation, with three receiving low-molecular-weight heparin (LMWH) followed by direct oral anticoagulants for a four-month course. One case only received 10 days of LMWH. Five out of six cases achieved complete resolution with three to five weeks of antibiotics, while one case had serious and complex complications and required admission to the PICU. This emphasizes the importance of considering differential diagnoses, especially during the COVID-19 era, and being aware of atypical symptoms of Lemierre's syndrome.

**Table 4:** Case reports PNA - peanut agglutinin, MIS-C - multisystem inflammatory syndrome in children, CTA - computed tomography angiography, IJV - internal jugular vein, PCR - polymerase chain reaction, CRO - ceftriaxone, MEM - meropenem, TZP - piperacillin-tazobactam, VAN - vancomycin, MTZ - metronidazole, AMC - amoxicillin-clavulanic acid

Author	Age/sex	Presentation	Initial diagnosis	COVID-19 test	Confirmatory Dx (# days since hospitalization)	Treatment	Anticoagulation	Outcome
Lima-Bernardes (2020) [5]	37/M	L-side neck pain, odynophagia, fever, dyspnea	Exudative tonsillitis, COVID-19 PNA	Neg x 3	Cervical CT: thrombosis of left IJV (7 days)	TZP x 5 weeks	Enoxaparin then Rivaroxaban for 4 months	Inflammatory markers back to normal in 2 weeks
Repper (2020) [7]	15/F	Sore throat, fever	Sepsis, MIS-C w/ b/l pulmonary infiltrates/edema, and L pleural effusion	Neg x 1	Neck/chest CTA: Monocclusive thrombus in the b/l IJV (4 days)	MEM followed by AMC x 4 weeks	Enoxaparin x 10 days	Discharged home after 10 days of hospitalization
Howley (2020) [10]	24/M	Headache, myalgia, LLL weakness, abdominal pain, anorexia	COVID-19 w/ superimposed bacteremia	Neg x 1	CTA: filling defect in L IJV (N/A)	CRO and MTZ	Therapeutic dose enoxaparin with following apixaban for 3 months	3 weeks in ICU and a further 2 weeks of ward-based rehab as an inpatient
Soares (2021) [8]	21/M	Sore throat, fever, chills, muscle aches, non-bloody emesis	COVID 19 PNA	Neg x 3 (PCR x 2, IgG x 1)	CT neck: non-occlusive thrombus in the R IJV (4 days)	TZP followed by CRO and VAN; discharged w/ CRO x 4 weeks	None	Symptoms resolved 2 weeks after discharge
Costa (2021) [9]	31/M	Fever, odynophagia, exertional dyspnea, myalgia, pleuritic chest pain	COVID-19 with a small amount of b/l pleural fluid	Neg x 3	Neck CT: occlusion of L cervical IJV (N/A)	TZP x 20 days	LMWH followed by Rivaroxaban for 4 months	Favorable outcome
Nguyen (2022)[6]	20/F	Dyspnea, pharyngitis	B/l multifocal, necrotizing PNA, pleural effusions with loculations, cavitary abscess	Neg x 4	Blood Cx: F. nucleatum (6 days)	CRO, VAN, and MTZ	None 2/2 thrombocytopenia	PICU admission

The mainstay of treatment is prolonged antibiotics based on sensitivity data. *F. necrophorum* typically does not exhibit beta-lactamase activity [11, 12], but there have been reports of beta-lactamase production and treatment failure [2, 13-16]. Therefore, empiric treatment with beta-lactamase-resistant antibiotics that have anaerobic activity, such as metronidazole, clindamycin, and piperacillin-tazobactam, is preferred. Ampicillin-sulbactam was discouraged due to the higher resistance rate [17]. Due to the scarcity of controlled clinical trials for determining the optimal antibiotic regimen, clinical decision-making in cases of Lemierre's syndrome primarily relied on culture sensitivity and case reports. Usually, a preferred treatment approach involves administering third-generation cephalosporins along with metronidazole for a period of two to six weeks. Despite providing appropriate antimicrobial treatment, the clinical response might be delayed, possibly due to challenges in antibiotic penetration into the fibrin clot.

The role of anticoagulation in such cases remains a topic of debate without a consensus. A generally accepted recommendation is to initiate anticoagulation only if thrombosis extends into the cerebral sinuses or symptoms persist even with sufficient antibiotic coverage. In these situations, the course should last three months, similar to provoked venous thrombosis [4, 18]. In the presented case, the patient responded well to antibiotic therapy without extensive thrombosis, and after weighing the risks and benefits, the decision was made to withhold anticoagulation. However, a recent systematic review of 14 cases showed that anticoagulation is effective and safe [19]. Surgical intervention is recommended for cases where abscess formation occurs, commonly observed in peritonsillar and lateral pharyngeal spaces. 

The prognosis of Lemierre's syndrome relies on awareness of the condition, maintaining a high level of suspicion, and initiating prompt antibiotic therapy. The overall mortality rate of Lemierre's syndrome is 9%, with a higher rate of 26% in elderly individuals [20]. However, these figures may underestimate the true incidence due to underdiagnosis or underreporting.

## Conclusions

This report highlighted a rare yet potentially life-threatening case of Lemierre's syndrome. Timely diagnosis, supported by radiographic evidence of thrombophlebitis or blood culture, along with prompt initiation of antibiotics, are essential for achieving better outcomes. Further prospective studies are necessary to address the existing clinical controversies surrounding this condition.
